# Neoadjuvant Chemotherapy Switch in Borderline Resectable/Locally Advanced Pancreatic Cancer

**DOI:** 10.1245/s10434-021-10991-2

**Published:** 2021-11-01

**Authors:** Roberto Alva-Ruiz, Lavanya Yohanathan, Jennifer A. Yonkus, Amro M. Abdelrahman, Lindsey A. Gregory, Thorvadur R. Halfdanarson, Amit Mahipal, Robert R. McWilliams, Wen Wee Ma, Christopher L. Hallemeier, Rondell P. Graham, Travis E. Grotz, Rory L. Smoot, Sean P. Cleary, David M. Nagorney, Michael L. Kendrick, Mark J. Truty

**Affiliations:** 1 Division of Hepatobiliary and Pancreas Surgery, Mayo Clinic, Rochester, MN USA; 2 Division of Medical Oncology, Mayo Clinic, Rochester, MN USA; 3 Department of Radiation Oncology, Mayo Clinic, Rochester, MN USA; 4 Department of Laboratory Medicine and Pathology, Mayo Clinic, Rochester, MN USA

## Abstract

**Background:**

Neoadjuvant chemotherapy (NAC) is an integral part of preoperative treatment for patients with borderline resectable/locally advanced (BR/LA) pancreatic ductal adenocarcinoma (PDAC). The identification of a chemotherapeutic regimen that is both effective and tolerable is critical for NAC to be of oncologic benefit. After initial first-line (FL) NAC, some patients have lack of response or therapeutic toxicities precluding further treatment with the same regimen; optimal decision making regarding this patient population is unclear. Chemotherapy switch (CS) may allow for a larger proportion of patients to undergo curative-intent resection after NAC.

**Methods:**

We reviewed our surgical database for patients undergoing combinatorial NAC for BR/LA PDAC. Variant histologic exocrine carcinomas, intraductal papillary mucinous neoplasm-associated PDAC, and patients without research consent were excluded.

**Results:**

Overall, 468 patients with BR/LA PDAC receiving FL chemotherapy were reviewed, of whom 70% (329/468) continued with FL chemotherapy followed by surgical resection. The remaining 30% (139/468) underwent CS, with 72% (100/139) of CS patients going on to curative-intent surgical resection. Recurrence-free survival (RFS) and overall survival (OS) were not significantly different between the resected FL and CS cohorts (30.0 vs. 19.1 months, *p *= 0.13, and 41.4 vs. 36.4 months, *p *= 0.94, respectively) and OS was significantly worse in those undergoing CS without subsequent resection (19 months, *p *< 0.0001). On multivariable analysis, carbohydrate antigen (CA) 19-9 and pathologic treatment responses were predictors of RFS and OS.

**Conclusion:**

CS in patients undergoing NAC for BR/LA pancreatic cancer does not incur oncologic detriment. The incorporation of CS into NAC treatment sequencing may allow a greater proportion of patients to proceed to curative-intent surgery.

Pancreatic ductal adenocarcinoma (PDAC) is an aggressive malignancy and the third most lethal cancer in the US.^1^ The all-stage 5-year relative survival rate is only 8% and has been relatively unchanged over the past decade due to the high proportion of patients with incurable metastatic disease at presentation.^2^ Although surgical resection is the only known curative option in the treatment of localized PDAC, patients with anatomically borderline resectable/locally advanced (BR/LA) PDAC are at high risk of positive margins, a well-established predictor of worse survival outcomes, as well as early metastatic recurrence with an upfront surgical strategy.^3,4^ Many studies have demonstrated the increased use of neoadjuvant chemotherapy (NAC) in the management of BR/LA PDAC prior to consideration of surgical resection.^5–8^ The advantages of a neoadjuvant chemotherapeutic approach include not only the identification of patients with chemoresistant disease who likely will not benefit from subsequent surgical resection but it may also potentially control or eradicate occult systemic disease and may increase the possibility of a subsequent margin-negative curative-intent resection.

As such, the majority of centers have now incorporated modern neoadjuvant combinatorial chemotherapy for patients with BR/LA tumors, per National Comprehensive Cancer Network guidelines.^9^ Although the ultimate goal of such a strategy is improved survival in this higher risk cohort of anatomically advanced but seemingly non-metastatic patients, this is predicated on the assumption that the NAC that is being administered is both demonstrably effective and tolerable. This is supported by the data suggesting those patients who are able to achieve significant pathologic treatment responses have improved oncologic outcomes.^10–13^

There are patients in whom first-line (FL) NAC results in either no objective responses, local or biochemical (carbohydrate antigen [CA] 19-9) progression, or who encounter significant treatment-related toxicities precluding further use of the same chemotherapy regimen. The optimal management of these patients is currently unknown. There are little data on the frequency, indications, and outcomes of patients who require chemotherapeutic switch (CS) from initial FL chemotherapy to a different chemotherapeutic regimen prior to curative-intent surgery. Our center was the first to describe such an approach and we have heavily incorporated this strategy prior to consideration of surgical resection since the introduction of modern combinatorial NAC regimens.^14,15^ The aim of this study was to report our cumulative high-volume institutional experience with CS in BR/LA pancreatic cancer patients.

## Methods

After Institutional Review Board approval was obtained, a retrospective review of a prospectively maintained PDAC surgical database for all patients undergoing NAC for BR/LA PDAC from 2009 to 2020 was performed. We specifically excluded patients with variant histologic exocrine carcinomas, intraductal papillary mucinous neoplasm-associated PDAC, and patients without research consent.

### Pathologic Evaluation

All patients had biopsy-confirmed pancreatic adenocarcinoma, with tissue collected via endoscopic ultrasound (EUS) fine needle aspiration. For any patients presenting with previous outside biopsies, those tissue blocks were formally interpreted by institutional gastrointestinal pathologists prior to initiating systemic chemotherapy. For patients undergoing resection after NAC, the College of American Pathology (CAP) protocols were used for tumor, margin, and nodal assessments.^16,17^ Pathologic treatment response was scored according to the CAP criteria: complete response, score 0 (no viable cancer cells); near complete response, score 1 (single/rare groups of cancer cells); partial response, score 2 (residual cancer with regression); poor/no response, score 3 (no tumor regression). For subsequent analyses, we grouped a score of 0 or 1 as major pathologic response and a score of 2 or 3 as minor pathologic response.

### Clinical Staging

All patients underwent standardized radiographic imaging at the time of diagnosis, which included non-contrast chest computed tomography (CT) and triple-phase (arterial, pancreatic, portal) abdomen/pelvis CT per pancreas protocol with axial, coronal, and sagittal reconstructions. All imaging was reviewed by both gastrointestinal radiologists and study surgeons, and patients were anatomically categorized as either BR/LA per Intergroup (Alliance) criteria based on the extent of extrapancreatic extension with venous and/or arterial involvement.^18^ In recent years, the majority of patients also underwent baseline positron emission tomography (PET) metabolic imaging (PET/CT or PET/MRI) prior to the initiation of induction chemotherapy. All patients underwent identical radiographic restaging following NAC after the completion of FL therapy and after CS prior to surgical resection. All patients had serum CA19-9 levels drawn at diagnostic baseline (after biliary stenting/drainage and just prior to the initiation of NAC) and serial levels were drawn throughout NAC. CA19-9 normalization (response) was determined at the conclusion of all systemic NAC and was dichotomized per previous methods.^15^

### Neoadjuvant Therapy

All patients received modern combinatorial NAC with either 5-fluorouracil-based chemotherapy (FOLFIRINOX/FOLFOX) or gemcitabine-based (gemcitabine/nab-paclitaxel) regimens, according to institutional protocols, with or without dose modifications, as deemed appropriate by treating oncologists. Chemotherapy was administered on a 2-week or 4-week cycle dependent on the specific regimen. Total cycles of chemotherapy were counted based on the total number of cycles administered during induction chemotherapy treatment. For those patients who underwent CS, we counted the total of all regimen cycles. Patients underwent routine restaging, typically every 2–3 months during induction chemotherapy treatment to evaluate for objective responses to treatment. Components of an objective response included clinical (improved pain, weight gain), biochemical (CA19-9 decrease), radiologic (decreased tumor size or less vascular involvement), or metabolic responses (decrease in tumoral PET avidity/viability). The majority of CSs were performed after the first restaging examination, typically after 2–3 months of FL chemotherapy, while a minority of patients underwent CS after the second or third restaging visits. Select patients received chemoradiation (CRT) following NAC and this was determined according to surgeon/oncology recommendations based on margin risk. CRT consisted of photon/proton external beam with a 50 Gy dose delivered in 25–28 daily fractions over 5 weeks, or a 45 Gy dose delivered in 15 fractions over 3 weeks, as per an institutional protocol with three-dimensional conformal or intensity-modulated techniques with concurrent radiosensitizing chemotherapy.

### Surgical Procedures

Patients were deemed candidates for surgical resection following NAC in the absence of metastatic disease after completion of therapy, technical feasibility for potentially achieving a negative margin resection with or without en bloc vascular and/or multivisceral resection and reconstruction as deemed by the operative surgeon, and patient fitness and condition permissible for surgery with general anesthesia. Radiologic anatomical downstaging was not a requirement for proceeding to resection if anatomy was favorable; however, other measures of objective response (clinical, biochemical, or metabolic response) were typically required as surrogates of chemotherapeutic treatment response. Surgical resections included pancreaticoduodenectomy, distal/subtotal pancreatectomy, and total pancreatectomy. Formal en bloc vascular resections were routinely performed with or without formal revascularization dependent on anatomical circumstances.

### Perioperative Outcomes

Postoperative complications were graded according to the standard Clavien–Dindo classification, as minor (grade 2 or lower) or major (grade 3A or higher) and reported as 90-day outcomes.^19^ Pancreas-specific complications such as delayed gastric emptying (DGE), postoperative pancreatic fistula (POPF), and post-pancreatectomy hemorrhage (PPH) were graded according to the International Study Group of Pancreatic Surgery (ISGPS) definitions, as clinically significant (grade B/C).^20^ Operative mortality was calculated as any death within 90 days after surgery, either as an inpatient or outpatient and either at our center or an outside facility. Postoperative surveillance occurred every 3–4 months for the first 3 years, every 6 months for years 4–5, and annually beyond 5 years. For those patients receiving follow-up at local facilities, laboratory and imaging records were faxed and uploaded into our electronic medical record and updated within our cancer registry. Follow-up phone calls to all living patients included in this cohort were performed every 3 months to confirm patient status.

### Statistical Analysis

Continuous variables were summarized using mean and standard deviation or median and interquartile range, while continuous variables were summarized using frequency and percentage. Differences between chemotherapy groups were analyzed using either a *t *test or Wilcoxon rank-sum test for continuous variables and Chi-square test or Fisher’s exact test for categorical variables. All statistical tests were two-sided and differences were considered significant when *p *< 0.05. Recurrence-free survival (RFS) was measured from the date of resection until detection of local, peritoneal, or distant metastases, or death, and overall survival (OS) was measured from the date of tissue diagnosis until death or unless otherwise specified. Median RFS and OS were estimated using the Kaplan–Meier method with 95% confidence intervals. Statistical analysis was performed using MedCalc Statistical Software version 20.0.0 (MedCalc Software Ltd, Ostend, Belgium; https://www.medcalc.org, 2020) or GraphPad 8.2.0 for Windows (GraphPad Software, San Diego, CA, USA; www.graphpad.com).

## Results

### Baseline Characteristics

**Fig. 1:**
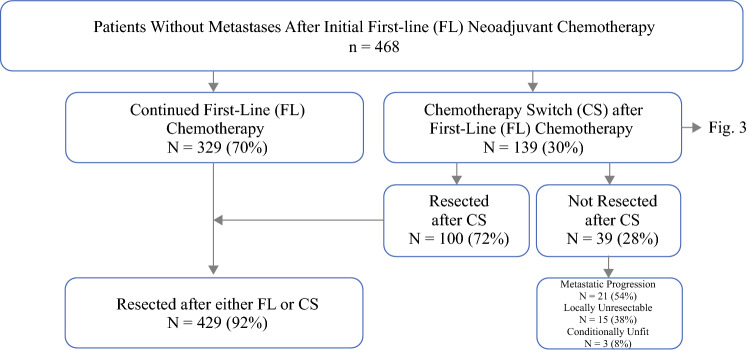
Study cohort schema

**Fig. 2:**
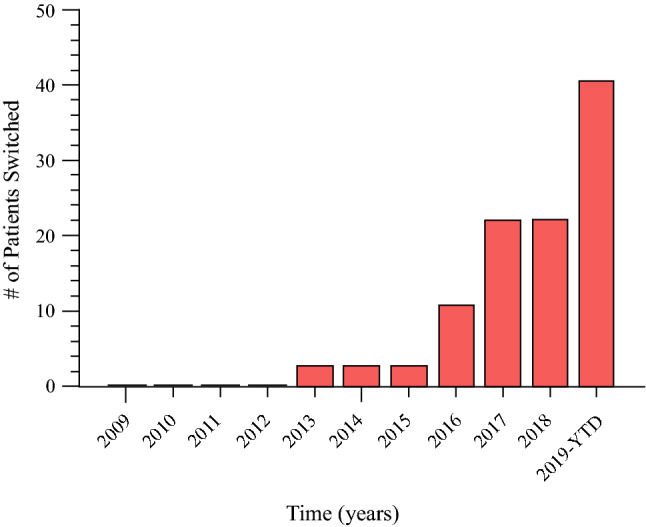
Chemotherapy switch over time. *YTD* year to date

**Fig. 3:**
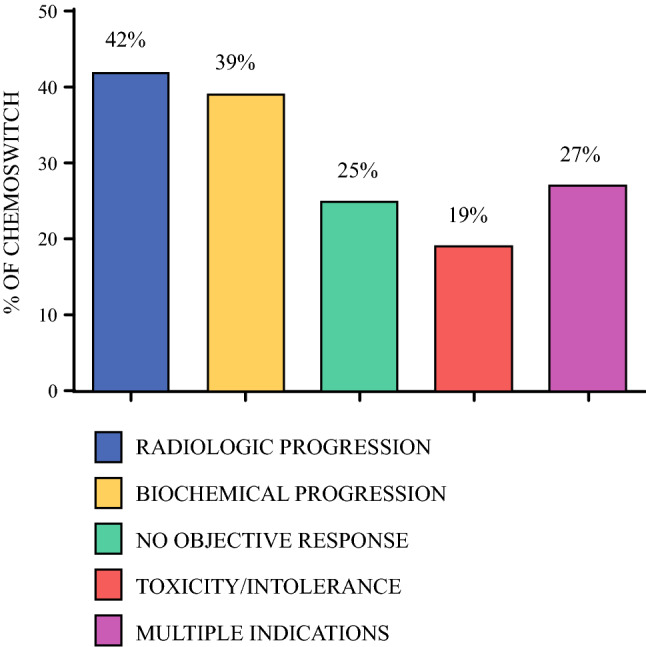
Frequency and indications for chemotherapy switch

After exclusions, we identified 468 patients with BR/LA PDAC who received initial FL NAC chemotherapy without development of metastatic disease on initial restaging examinations (Fig. 1). Of this initial cohort, 70% (329/468) continued with the FL chemotherapy regimen and subsequently underwent surgical resection. The median number of chemotherapy cycles in the FL cohort was six, the median number of FL 5-fluorouracil-based cycles was six, and the median number of FL gemcitabine-based cycles was four. The remaining 30% (139/468) of patients underwent chemotherapy switch (CS) following initial FL chemotherapy with a median of four cycles of initial FL chemotherapy. The number of patients undergoing CS steadily increased over the study period (Fig. 2). Of the 139 patients who underwent CS, 89% (124/139) switched from 5-fluorouracil-based chemotherapy to gemcitabine-based chemotherapy, while 11% (15/139) switched from gemcitabine-based chemotherapy to 5-fluorouracil-based chemotherapy (Fig. 3). Indications for CS included non-metastatic radiologic progression in 42% (58/139) of patients, biochemical (CA19-9) progression in 39% (54/139) of patients, no objective response in 25% (35/139) of patients, and chemotherapy toxicity/intolerance in 19.4% (27/139) of patients. There were 38 patients (27%) with more than one indication. The median number of total chemotherapy cycles in the CS cohort was eight. Of the patients undergoing CS, 28% (39/139) did not undergo subsequent resection for the following reasons: 54% (21/39) of patients developed metastatic progression after CS, 38% (15/39) had anatomy precluding resectability despite CS, and 8% (3/39) of patients were deemed conditionally unfit for resection. Overall, 72% (100/139) of patients who underwent CS underwent surgical resection.

Perioperative and oncologic outcomes were assessed comparing patients undergoing successful resection following either FL or CS. Patient cohort demographics and variables of the 429 patients who underwent resection after FL (329 patients) or CS (100 patients) are shown in Table 1.

**Table 1:** Patient cohort demographics and variables

Variable	First-line (*n =* 329)	Chemotherapy switch (*n =* 100)	*p *value
Sex			
Male	169 (52)	51 (51)	0.9487
Female	160 (48)	49 (49)	
Race/ethnicity			
White	299 (90.9)	88 (88)	0.605
Black	3 (0.9)	2 (2)	
Asian	9 (2.7)	3 (3)	
Hispanic	8 (2.4)	4 (4)	
American Indian/Alaskan Native	3 (0.9)	1 (1)	
Unknown/not disclosed/other	7 (2.1)	2 (2)	
Age at surgery, years			0.0588
Mean (SD)	64.7	62.6	
Median	64.9	62.3	
Q1, Q3	58.6, 71.4	57.8, 68.2	
Median CA19-9 at diagnosis, U/mL	156	161.5	0.8980
CA19-9 elevated at diagnosis			
Yes	261 (79)	78 (78)	0.7749
No	68 (21)	22 (22)	
First-line chemotherapy type			0.0092
5-fluorouracil-based	254 (77)	89 (89)	
Gemcitabine-based	75 (23)	11 (11)	
Total no. of chemotherapy cycles [mean/median]	6.0/6.0	8.7/8.0	0.0001
More than six chemotherapy cycles			< 0.0001
Yes	107 (33)	73 (73)	
No	222 (67)	27 (27)	
CA19-9 normalization			0.1247
Yes	182 (55)	64 (64)	
No	147 (45)	36 (36)	
Chemoradiation			0.0062
Yes	264 (80)	92 (92)	
No	65 (20)	8 (8)	
Pancreatectomy type			< 0.0001
Total pancreatectomy	40 (12)	37 (37)	
Distal pancreatectomy	77 (23)	21 (21)	
Whipple	212 (65)	42 (42)	
Open resection			0.0061
Yes	273 (83)	94 (94)	
No	56 (17)	6 (6)	
Vascular resection			
Yes	199 (60)	60 (60)	0.9307
No	130 (40)	40 (40)	
Multivisceral organ resection			
Yes	54 (16)	35 (35)	0.0001
No	275 (84)	65 (65)	
Estimated blood loss, mL			0.0333
Mean (SD)	846 (780)	1052 (1027)	
Median	550	700	
Q1, Q3	335, 1093	400, 1450	
Range	20–4900	100–6400	
Perioperative PRBC transfusion			0.0027
Yes	104 (32)	48 (48)	
No	225 (68)	52 (52)	
Resection margin status			0.5670
Positive	29 (9)	7 (7)	
Negative	300 (91)	93 (93)	
No. of lymph nodes removed			0.0058
Mean (SD)	20 (9)	23 (10)	
Median	18	22	
Q1, Q3	14, 24	15, 29	
Range	1–54	1–47	
Positive lymph nodes			0.3323
Yes	92 (28)	33 (33)	
No	237 (72)	67 (67)	
Pathological treatment response score			0.4493
0	29 (9)	7 (7)	
1	74 (22)	23 (23)	
2	160 (49)	56 (56)	
3	66 (20)	14 (14)	
Major pathologic response, 0 or 1			0.9018
Yes	103 (31)	30 (30)	
90-day operative mortality			0.9471
Yes	17 (5)	5 (5)	
No	312 (95)	95 (95)	
Any complications			0.3426
Yes	215 (65)	60 (60)	
No	114 (35)	40 (40)	
Major (higher than grade III)	97 (29)	37 (37)	
DGE			0.7643
Yes	58 (18)	16 (16)	
No	271 (82)	84 (84)	
POPF			0.7247
Yes	38 (12)	13 (13)	
No	291 (88)	87 (87)	
PPH			0.9999
Yes	29 (9)	9 (9)	
No	300 (91)	91 (91)	
Length of stay, days			0.0040
Mean (SD)	10.9 (11)	15.3 (20)	
Median	8	10	
Q1, Q3	6, 12	7, 17.75	
Range	0–80	4–187	
Readmission			0.5172
Yes	84 (25)	29 (29)	
No	245 (75)	71 (71)	
Adjuvant chemotherapy			0.2764
Yes	125 (38)	32 (32)	
No	204 (62)	68 (68)	
Any recurrence			0.1920
Yes	166 (50)	57 (57)	
No	163 (50)	43 (43)	
Distant recurrence	110 (33)	41 (41)	0.5050
Peritoneal recurrence	31 (19)	16 (16)	0.1860
Local recurrence	46 (9)	13 (13)	0.4904
Alive at last follow-up			0.3577
Yes	144 (44)	49 (49)	
No	185 (56)	51 (51)	

Data are expressed as *n* (%) unless otherwise specified

*SD* standard deviation, *Q1* first quartile, *Q3* third quartile, *CA* carbohydrate antigen, *PRBC* packed red blood cells, *DGE* delayed gastric emptying, *POPF* postoperative pancreatic fistula, *PPH* post-pancreatectomy hemorrhage

### Patient Demographics

There were no significant differences in sex, age, or baseline CA19-9 levels in either the FL or CS cohorts. CS patients received a higher number of cycles of chemotherapy compared with the FL group (8 vs. 6, *p *= 0.0001) and were more likely to receive CRT after induction chemotherapy treatment than FL patients (92% vs. 80.2%, *p *= 0.005). Overall, the FL and CS groups had a similar proportion of patients with normalization of CA19-9 levels after completion of all NAC (55.3% vs. 64%, *p *= 0.13).

Patients in the FL cohort were more likely to have laparoscopic resections (17% vs. 6%, *p *= 0.006), while total pancreatectomy was more common in CS patients and pancreaticoduodenectomy was more common in FL patients. There was no difference in the rate of en bloc vascular resections between the FL and CS cohorts (60% vs. 60%, *p *= 0.93), however multivisceral resection was more common in the CS patients (16.4% vs. 35%, *p *= 0.0001). The median estimated blood loss and perioperative transfusion was lower in the FL group compared with the CS group (550 mL vs. 700 mL, *p *= 0.03; 32% vs. 48%, *p *= 0.003). Perioperative outcomes were similar between groups, with similar 90-day major morbidity (29% vs. 37%, *p *= 0.34), 90-day operative mortality (5.2% vs. 5.0%, *p *= 0.95), and 30-day readmission rate (25% vs. 29%, *p *= 0.52) between the FL and CS cohorts, respectively. The median length of stay in patients following FL chemotherapy was shorter than CS (8 vs. 10 days, *p *= 0.004).

The rates of margin-positive resections (8.8% vs. 7%, *p *= 0.57) and lymph node positivity (28% vs. 33%, *p *= 0.33) were similar, as were major pathologic treatment response scores (CAP score 0/1) [31.3% vs. 30%, *p *= 0.45], between the FL and CS cohorts, respectively. There was no difference in receipt of adjuvant chemotherapy between groups, i.e. 38% of FL patients compared with 32% in the CS group, which was not significantly different (*p *= 0.28).

**Fig. 4:**
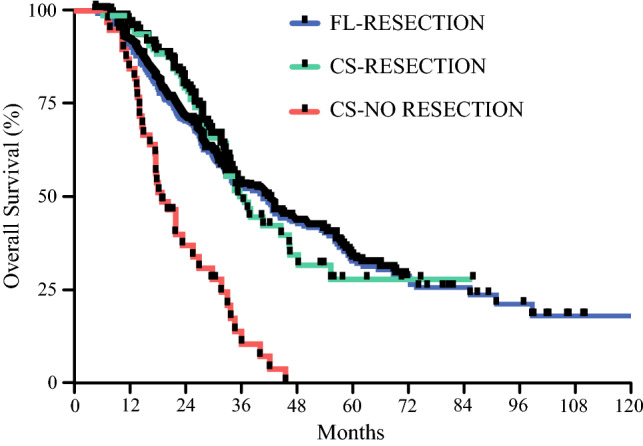
Overall survival for FL-resection, CS-resection, and CS-no resection. *FL* first-line, *CS* chemotherapy switch

Median follow-up for the resected cohort was 29.5 months, and there was no difference in recurrence rate between the FL and CS groups (50.4% vs. 57%, *p *= 0.19). On subset recurrence analysis, there was no difference in local, peritoneal, or distant recurrence between both cohorts, with distant metastases being the most common recurrence pattern in both groups. At the time of last follow-up, 46% of the entire cohort was alive, which was similar between groups (*p *= 0.36). There was no statistically significant difference in the median RFS between the FL and CS cohorts (30 vs. 19.1 months, *p *= 0.1332). Similarly, there was no significant difference in median OS between FL and CS patients (41.4 vs. 36.4 months, *p *= 0.9391). The median OS for those patients who underwent CS but did not ultimately undergo resection was 19 months, which was significantly worse than those patients who did undergo resection after either FL or CS (*p ≤* 0.0001) (Fig. 4).

**Fig. 5:**
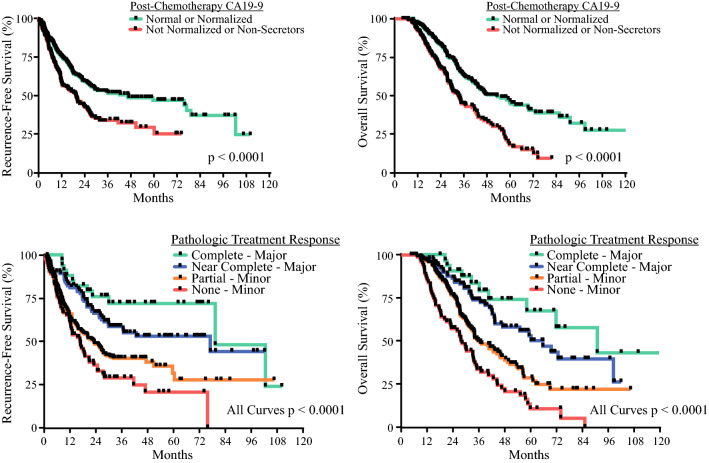
Recurrence-free survival and overall survival for CA19-9 normalization and pathologic treatment response. *CA* carbohydrate antigen

Univariate predictors of RFS and OS are shown in Table 2. CS was not associated with detrimental survival outcomes. Tumor grade, lymph node status, CA19-9 response, and pathologic treatment response were associated with RFS, while chemotherapy duration, perioperative blood transfusion, multivisceral resection, major complications, tumor grade, margin status, lymph node status, CA19-9 response, pathologic treatment response, and adjuvant chemotherapy were associated with OS. On multivariate analysis (Table 3), chemotherapy duration, margin status, major complications, and adjuvant therapy were independent predictors of OS only, while CA19-9 response and pathologic treatment response were independent predictors of both RFS and OS (Fig. 5)

**Table 2:** Univariate analysis of variables associated with recurrence-free survival and overall survival

Variable	Recurrence-free survival		Overall survival	
	HR (95% CI)	*p* value	HR (95% CI)	*p* value
Sex				
Male	1.0 (reference)		1.0 (reference)	
Female	0.855 (0.652–1.121)	0.256	0.966 (0.735–1.270)	0.806
Chemotherapy switch				
No	1.0 (reference)		1.0 (reference)	
Yes	1.231 (0.897–1.690)	0.197	1.022 (0.725–1.440)	0.903
More than six chemotherapy cycles				
No	1.0 (reference)		1.0 (reference)	
Yes	1.085 (0.944–1.246)	0.251	0.696 (0.520–0.931)	0.015
Chemoradiation				
No	1.0 (reference)		1.0 (reference)	
Yes	0.978 (0.683–1.4)	0.901	1.026 (0.704–1.497)	0.893
Operation type				
Total pancreatectomy	1.0 (reference)		1.0 (reference)	
Distal pancreatectomy	1.210 (0.792–1.849)	0.378	1.272 (0.809–2.002)	0.298
Pancreaticoduodenectomy	0.890 (0.613–1.293)	0.541	1.033 (0.692–1.542)	0.874
Approach				
Open	1.0 (reference)		1.0 (reference)	
MIS	1.123 (0.784–1.608)	0.526	1.097 (0.776–1.549)	0.601
Perioperative PRBC transfusion				
No	1.0 (reference)		1.0 (reference)	
Yes	1.274 (0.962–1.689)	0.092	1.331 (1.004–1.765)	0.047
Tumor grade				
0^a^	1.0 (reference)		1.0 (reference)	
1	2.038 (0.907–4.583)	0.085	1.755 (0.788–3.907)	0.169
2	3.097 (1.514–6.332)	0.002	2.958 (1.446–6.050)	0.003
3/4	3.419 (1.609–7.267)	0.001	2.904 (1.362–6.189)	0.006
Lymphovascular invasion				
No	1.0 (reference)		1.0 (reference)	
Yes	1.117 (0.774–1.614)	0.553	1.104 (0.767–1.589)	0.594
Margin status				
Negative	1.0 (reference)		1.0 (reference)	
Positive	1.188 (0.741–1.907)	0.474	2.132 (1.410–3.225)	< 0.001
Lymph node status				
Negative	1.0 (reference)		1.0 (reference)	
Positive	1.476 (1.106–1.970)	0.008	1.494 (1.119–1.994)	0.006
Pathologic treatment response				
Minor	1.0 (reference)		1.0 (reference)	
Major	0.474 (0.344–0.654)	< 0.001	0.398 (0.285–0.557)	< 0.001
Any vascular resection				
No	1.0 (reference)		1.0 (reference)	
Yes	1.072 (0.814–1.413)	0.621	1.128 (0.853–1.492)	0.398
Multivisceral organ resection				
No	1.0 (reference)		1.0 (reference)	
Yes	1.226 (0.873–1.721)	0.240	1.512 (1.073–2.131)	0.018
CA19-9 elevation at diagnosis				
No	1.0 (reference)		1.0 (reference)	
Yes	1.186 (0.837–1.680)	0.337	1.105 (0.774–1.579)	0.582
CA19-9 normalization				
No	1.0 (reference)		1.0 (reference)	
Yes	0.607 (0.461–0.798)	< 0.001	0.510 (0.386–0.673)	< 0.001
Readmission				
No	1.0 (reference)		1.0 (reference)	
Yes	0.964 (0.706–1.316)	0.816	1.087 (0.793–1.489)	0.605
Any complication				
No	1.0 (reference)		1.0 (reference)	
Yes	1.046 (0.789–1.386)	0.757	1.088 (0.818–1.448)	0.562
Higher than grade III complication				
No	1.0 (reference)		1.0 (reference)	
Yes	1.188 (0.883–1.599)	0.255	1.360 (1.005–1.841)	0.046
POPF				
No	1.0 (reference)		1.0 (reference)	
Yes	0.968 (0.627–1.493)	0.882	1.160 (0.763–1.762)	0.488
PPH				
No	1.0 (reference)		1.0 (reference)	
Yes	1.221 (0.733–2.034)	0.443	1.554 (0.969–2.495)	0.068
DGE				
No	1.0 (reference)		1.0 (reference)	
Yes	0.887 (0.606–1.299)	0.539	0.966 (0.665–1.401)	0.854
Adjuvant therapy				
No	1.0 (reference)		1.0 (reference)	
Yes	0.982 (0.745–1.295)	0.899	0.750 (0.563–0.998)	0.048

*HR* hazard ratio, *CI* confidence interval, *MIS* minimally invasive surgery, *CA* carbohydrate antigen, *PRBC* packed red blood cells, *POPF* postoperative pancreatic fistula, *PPH* post-pancreatectomy hemorrhage, *DGE* delayed gastric emptying

^a^Grade 0—complete pathologic response

**Table 3:** Multivariate analysis of variables associated with recurrence-free survival and overall survival

Variable	Recurrence-free survival		Overall survival	
	HR (95% CI)	*p *value	HR (95% CI)	*p *value
Tumor grade				
0^a^	1.0 (reference)		1.0 (reference)	
1	1.593 (0.701–3.621)	0.266	1.245 (0.549–2.825)	0.600
2	1.801 (0.825–3.935)	0.140	1.308 (0.587–2.914)	0.512
3/4	2.142 (0.957–4.796)	0.064	1.536 (0.671–3.516)	0.310
Lymph node status				
No	1.0 (reference)		1.0 (reference)	
Yes	1.172 (0.870–1.578)	0.296	1.238 (0.899–1.704)	0.192
Perioperative PRBC transfusion				
No	–		1.0 (reference)	
Yes	–	–	1.161 (0.848–1.591)	0.352
Margin status				
Negative	–		1.0 (reference)	
Positive	–	–	1.916 (1.222–3.006)	0.005
Pathologic treatment response				
Minor	1.0 (reference)		1.0 (reference)	
Major	0.617 (0.421–0.904)	0.013	0.539 (0.354–0.829)	0.004
Multivisceral organ resection				
No	–		1.0 (reference)	
Yes	–	–	1.357 (0.912–2.020)	0.132
CA19-9 normalization				
No	1.0 (reference)		1.0 (reference)	
Yes	0.677 (0.512–0.896)	0.006	0.577 (0.433–0.769)	< 0.001
More than six chemotherapy cycles				
No	–		1.0 (reference)	
Yes	–	–	0.615 (0.443–0.851)	0.003
Adjuvant therapy				
No	–		1.0 (reference)	
Yes	–	–	0.603 (0.446–0.816)	0.001
Higher than grade III complication				
No	–		1.0 (reference)	
Yes	–	–	1.495 (1.086–2.058)	0.014

*HR* hazard ratio, *CI* confidence interval, *CA* carbohydrate antigen, *PRBC* packed red blood cells

^a^ Grade 0—complete pathologic response

## Discussion

In this study of 468 patients with BR/LA PDAC undergoing FL NAC, we demonstrated that a substantial proportion of patients (30%) required a chemotherapeutic switch due to a variety of indications, primarily due to ineffective treatment. Of those who underwent CS, a majority were able to achieve therapeutic benefit (72%), proceeding to curative-intent surgical resection after CS. We found no major preoperative or perioperative differences between those undergoing resection after FL NAC and those who required CS. Furthermore, pathologic outcomes and, most importantly, survival outcomes were not significantly different between cohorts, suggesting that such a CS strategy has no oncologic detriment and can potentially enable surgical salvage in a considerable proportion of patients.

While surgical resection has long been considered important for the long-term survival of localized but non-metastatic PDAC, surgical resection alone is not sufficient given the overwhelming probability of postoperative recurrence. The use of neoadjuvant therapy has been increasingly adopted as the primary treatment strategy for BR/LA PDAC to address the high distant failure rates and high positive margin rates historically seen with a surgery-first approach for such tumors.^21^ The central tenet to a neoadjuvant strategy is the selection and use of both a demonstrably ‘effective’ and clinically ‘tolerable’ chemotherapy regimen.^22–25^ In order to determine whether our initial chemotherapeutic regimen of choice meets these criteria, patients are routinely and periodically evaluated for treatment toxicities and objective responses (clinical, radiologic, biochemical, metabolic) during this phase of treatment, typically every 2 months. In those patients in whom we are able to establish both objective responses and tolerance, we typically continue FL therapy. There is no consensus on what should be done for those patients without objective responses or who develop significant treatment toxicities precluding further FL chemotherapy administration.

Our center has been implementing a chemotherapeutic switch strategy for many years, since the availability of dual 5-fluorouracil and gemcitabine-based regimens for such situations.^14,15^ Prior to this current study, outcomes of patients who were salvaged by second-line chemotherapy in the neoadjuvant setting were not well-described and were limited to case reports.^26,27^ We have found that such a strategy allows for significant surgical salvage of patients with either chemoresistance or therapeutic toxicity to FL therapy. These findings highlight the biological heterogeneity that exists in all PDAC, in which some tumors are responsive to one regimen or the other and sometimes chemoresistant to both. We found no significant major demographic or clinical differences between those undergoing resection after FL chemotherapy compared with those after CS. One-third of patients ultimately did not undergo resection after CS, primarily due to metastatic progression after CS, again highlighting one of the advantages of neoadjuvant therapy as a negative selection tool to determine which patients may not benefit from surgical resection, as the majority of patients likely harbor occult metastases at the time of resection.^28^ In contrast, nearly three-quarters of patients were able to either better tolerate or achieve subsequent objective responses to CS, leading to curative-intent resection further supporting the use of such a strategy.

From an operative standpoint, we showed that there is no difference in major morbidity or mortality despite patients undergoing CS being more likely to require more extensive multivisceral resections. In addition, there was no significant difference in the need for vascular resection and reconstruction, morbidity, or readmission rate, and no difference in the margin positivity rate between both cohorts. Importantly, we demonstrated CS has no detrimental effect on postoperative RFS or OS, with those patients undergoing CS with surgery having markedly improved survival compared with those with CS without surgery, emphasizing the potential surgical salvage benefit with this strategy. Our group has previously shown that extended duration of chemotherapy and modifications of initial chemotherapeutic treatment by either extending the cycle duration or consideration for CS may significantly influence postoperative survival.^15^ Other groups have shown similar response factors that may markedly affect oncologic outcomes.^29–31^ This is thought to be primarily due to the fact that both NAC effectiveness and duration are closely correlated to pathologic treatment response, one of the major single independent predictors of survival in numerous studies.^10,12,32,33^

While this study represents the largest series of patients undergoing NAC switch, it has significant limitations due to its retrospective design. First, we do not know how many patients who began FL NAC progressed or expired prior to returning to our center for restaging or went elsewhere for their care, thus the initial dropout rate for FL NAC is unknown in the absence of a prospective trial. Second, although our data show that a high proportion of BR/LA patients were able to undergo resection after either FL or CS, this high rate of resection compared with other centers is likely due to our more liberal surgical resection criteria through the utilization of more extensive en bloc major vascular resections. Third, our institutional criteria for response is not dependent on traditional radiologic (CT/MR) anatomic downstaging, which further increases the proportion of patients who are candidates for resection. Finally, weekly chemotherapeutic dose adjustments were not accounted for in this study, and chemotherapy received was measured by receipt of cycle only and not chemotherapy intensity.

Despite these limitations, the presented results contribute to the understanding and utility of appropriate neoadjuvant treatment sequencing for anatomically borderline resectable and locally advanced pancreatic adenocarcinoma and the utility of altering the initial chemotherapeutic plan based on responses and tolerance. Neoadjuvant CS is not only feasible in patients with BR/LA PDAC but can potentially salvage a significant proportion of patients who may otherwise be deemed inoperable. Based on this study, we suggest that CS be considered as a significantly important neoadjuvant strategy in all patients with BR/LA pancreatic cancer with inadequate response or intolerance to FL chemotherapy.
